# Identifying influencing factors associated with sleep quality in undergraduates based on partial least squares regression and XGBoost

**DOI:** 10.3389/fpsyg.2025.1732946

**Published:** 2026-01-12

**Authors:** Yuchen Xie, Yuan Chen, Yaohui Han, Shilei Zhai, Lishun Xiao, Dehui Yin, Yansu Chen

**Affiliations:** 1 Publicity Department, Xuzhou Medical University, Xuzhou, Jiangsu, China; 2 School of Public Health, Xuzhou Medical University, Xuzhou, Jiangsu, China

**Keywords:** multicollinearity, nonlinear pattern, partial least squares regression, sleep quality, XGBoost

## Abstract

**Background:**

This study aimed to identify the influencing factors associated with sleep quality among undergraduates in Jiangsu, China, and to explore their complex relationships.

**Methods:**

A cross-sectional survey was conducted online between October and November 2022. A total of 7,062 valid participants (aged 20.1 ± 1.3 years) were included, and a complete case analysis was performed. Sleep quality was assessed using the Pittsburgh Sleep Quality Index (PSQI), with a score exceeding 7 denoting poor sleep quality. To overcome the limitations of traditional statistical methods in handling multicollinearity and capturing complex, nonlinear associations, Partial Least Squares Regression (PLSR) was used to quantify linear relationships between influencing factors and quantitative PSQI scores, while the eXtreme Gradient Boosting (XGBoost) model and SHapley Additive exPlanations (SHAP) analysis were utilized to identify nonlinear influencing factors and their interactions associated with binary classification of sleep quality.

**Results:**

The prevalence of poor sleep quality was 26.5% (95%CI: 25.5 to 27.6%). Mental health status was the most influencing factor, and those with good mental health had better sleep quality (*β* = −0.853, 95%CI: −1.079 to −0.627, *p* < 0.001). Smartphone dependence (MPAI: *β* = 0.043, 95%CI: 0.038 to 0.048, *p* < 0.001) and a noisy dormitory environment (*β* = 0.627, 95%CI: 0.420 to 0.835, *p* < 0.001) were significantly associated with poorer sleep quality, whereas psychological resilience (CD-RISC: *β* = −0.017, 95%CI: −0.023 to −0.012, *p* < 0.001) emerged as a negatively associated factor. Other significant associations were found for smoking (*β* = 0.496, 95%CI: 0.276 to 0.716, *p* = 0.002), drinking (*β* = 0.594, 95%CI: 0.420 to 0.768, *p* < 0.001), and harmonious interpersonal relationships (*β* = −0.172, 95%CI: −0.251 to −0.094, *p* = 0.002). The XGBoost model showed good discriminative performance (AUC = 0.818, 95%CI: 0.795 to 0.841). SHAP analysis revealed nonlinear patterns, such as a U-shaped relationship between BMI and sleep quality.

**Conclusion:**

The integrated PLSR-XGBoost framework effectively handled multicollinearity without discarding variables and provided a more comprehensive understanding from both linear and nonlinear perspectives. The findings support the development of comprehensive and tailored interventions targeting specific influencing factors, such as mental health support, dormitory noise management, smartphone use modification, and resilience-building programs, offering an empirical foundation for sleep health promotion initiatives in university settings.

## Introduction

Sleep has been recognized as a fundamental physiological necessity, exerting profound effects on physical health, emotional well-being, and cognitive functions (Mukherjee et al., 2024). For undergraduates, who are at a critical stage of development, high-quality sleep is essential not only for health and cognition but also for academic success and overall development (Cheng and Carroll, 2020). However, the accelerating pace of society and widespread use of electronic devices have made poor sleep quality an increasingly prevalent issue in this population, with rising rates of insomnia, late-night habits, and sleep deprivation (Lu et al., 2024; Tao et al., 2024; Zhao and Zhang, 2024).

Epidemiological studies consistently report high rates of poor sleep quality in undergraduates across diverse regions, including 57.6% in a 60-country study (Babicki et al., 2023), over 60% in the U.S. (Mbous et al., 2022), and 34.2% among females in Norway (Sivertsen et al., 2019). In Africa, a systematic review of university students found a pooled prevalence of 63.31% (Nakie et al., 2024). Similarly, studies from Hong Kong (68.6%), Chongqing (49.7%), and Jilin (31.0%) highlight the issue in China (Sing and Wong, 2010; Li et al., 2020a; Wang et al., 2023). Poor sleep in students is linked to fatigue, impaired concentration, and increased risks of mental health disorders and chronic diseases over time (Seow et al., 2020), including cardiovascular disease, diabetes, and even elevated mortality risk (Del Brutto et al., 2024; Rogers et al., 2024). Identifying key influencing factors is therefore essential for effective intervention. Known contributors include academic and employment pressure (Zhao and Zhang, 2024), excessive electronic device use (Zhu et al., 2023; Tao et al., 2024), poor dietary habits, and disruptive dormitory environments (Sexton-Radek and Hartley, 2013; Faris et al., 2022).

Despite extensive research on undergraduate sleep, key limitations persist. First, large-scale studies are scarce in Jiangsu Province, a major higher-education hub with distinct socio-academic conditions, limiting locally relevant insights and generalizability within China. Second, traditional statistical methods (logistic, ridge, or Lasso regression) are ill-suited for such multifactorial data: they either become unstable under multicollinearity or obscure variable contributions and cannot capture nonlinear relationships or complex interactions. To address these gaps, we conducted a large-scale survey in Jiangsu using the Pittsburgh sleep quality index (PSQI) and introduced a novel dual-model framework combining partial least squares regression (PLSR) and extreme gradient boosting (XGBoost). PLSR retained all variables in the presence of multicollinearity to quantify linear associations, while XGBoost captured nonlinear influencing factors and their associations with sleep quality. This integrated approach comprehensively examines demographic, familial, behavioral, psychological, academic, environmental, and digital lifestyle factors affecting sleep quality.

## Materials and methods

### Participants

A cross-sectional online survey was conducted via the Wenjuanxing platform^1^ between October and November 2022. The questionnaire link and QR code were distributed to undergraduates through academic staff and WeChat groups. An introductory statement explained the study’s purpose, emphasized voluntary participation and the right to withdraw at any time, and guaranteed data anonymity and confidentiality. To ensure data quality, the questionnaire was pilot-tested among 50 students; items with comprehension rates below 90% were revised. The required sample size was calculated using the formula for cross-sectional prevalence studies: *n* = *z*^2^*pq*/*d*^2^. With *α* = 0.05 (two-sided, *z* = 1.96), an expected sleep-disorder prevalence of 24% (*p* = 0.24, *q* = 0.76), and a desired absolute precision of 2.5 percentage points (*d* = 0.025), the minimum sample size was 1,123. After allowing for a 10% invalid response rate, the target sample size was set at ≥ 1,236. In total, 7,062 valid questionnaires were collected after excluding incomplete responses, which far exceeded the minimum requirement and ensures high precision in the estimated prevalence. Therefore, a complete case analysis was performed in the present study.

### Questionnaire content

The questionnaire assessed demographics (gender, age, height, weight, grade, native place), family background (parental education level, family economic situation, monthly living expenses), lifestyle habits (smoking, drinking, physical exercise frequency), academic and employment pressures, dormitory environment (noise, lighting, and hygiene conditions) and interpersonal relations, PSQI, mobile phone addiction index (MPAI), Connor-Davidson resilience scale (CD-RISC), and other variables as listed in Table 1.

**Table 1:** The basic demographic characteristics across sleep quality of undergraduates in Jiangsu, China.

Variables	Overall 7,062 (100)	Good 5,189 (73.48)	Poor 1,873 (26.52)	$t/\chi^{2}$	*P* values
Age (year)	18.72 ± 0.95	18.67 ± 0.93	18.83 ± 1.00	5.99	**<0.001** ^**#**^
Height (cm)	167.54 ± 8.4	167.64 ± 8.39	167.27 ± 8.44	1.60	0.1096^#^
Weight (kg)	60.53 ± 12.66	60.71 ± 12.66	60.04 ± 12.67	1.97	**0.0491** ^**#**^
BMI (kg/m^2^)	21.48 ± 3.75	21.52 ± 3.73	21.37 ± 3.79	1.42	0.1545^#^
PSQI	5.64 ± 3.59	3.92 ± 2.00	10.40 ± 2.59	98.19	**<0.001**
Subjective sleep quality	0.91 ± 0.63	0.74 ± 0.52	1.37 ± 0.68	36.87	**<0.001**
Sleep latency	1.22 ± 1.41	0.72 ± 0.95	2.61 ± 1.54	49.99	**<0.001**
Sleep duration	0.94 ± 0.88	0.71 ± 0.74	1.59 ± 0.90	37.90	**<0.001**
Habitual sleep efficiency	0.89 ± 1.37	0.46 ± 1.08	2.09 ± 1.38	46.16	**<0.001**
Sleep disturbances	0.97 ± 0.65	0.79 ± 0.55	1.45 ± 0.65	39.07	**<0.001**
Use of sleeping medication	0.11 ± 0.43	0.05 ± 0.26	0.29 ± 0.70	14.66	**<0.001**
Daytime dysfunction	0.60 ± 0.65	0.46 ± 0.56	1.00 ± 0.71	33.36	**<0.001** ^**#**^
CD-RISC	85.64 ± 18.18	87.17 ± 18.38	81.40 ± 16.89	12.39	**<0.001**
Self-improvement	28.58 ± 6.04	29.09 ± 6.11	27.17 ± 5.61	12.39	**<0.001**
Tenacity	43.77 ± 10.00	44.58 ± 10.11	41.51 ± 9.33	11.94	**<0.001**
Optimism	13.29 ± 3.11	13.50 ± 3.11	12.72 ± 3.03	9.54	**<0.001**
MPAI	44.94 ± 13.55	43.33 ± 13.22	49.41 ± 13.45	16.98	**<0.001** ^**#**^
Inability to control craving	19.20 ± 6.11	18.48 ± 5.88	21.20 ± 6.28	16.85	**<0.001** ^**#**^
Withdrawal and escape	7.84 ± 3.46	7.48 ± 3.38	8.83 ± 3.50	14.65	**<0.001** ^**#**^
Anxiety and feeling lost	8.97 ± 3.21	8.71 ± 3.20	9.70 ± 3.13	11.56	**<0.001** ^**#**^
Productivity loss	8.93 ± 3.05	8.66 ± 3.03	9.68 ± 2.95	12.61	**<0.001** ^**#**^
Gender: Female	4,976 (70.46)	3,628 (69.92)	1,348 (71.97)	2.70	0.100
Male	2086 (29.54)	1,561 (30.08)	525 (28.03)		
Native place: Rural	4,294 (60.80)	3,114 (60.01)	1,180 (63.00)	5.00	**0.020**
Urban	2,768 (39.20)	2075 (39.99)	693 (37.00)		
Only child: No	4,366 (61.82)	3,150 (60.71)	1,216 (64.92)	10.00	**0.001**
Yes	2,696 (38.18)	2039 (39.29)	657 (35.08)		
Smoking: No	6,724 (95.21)	4,990 (96.16)	1734 (92.58)	38.00	**<0.001**
Yes	338 (4.79)	199 (3.84)	139 (7.42)		
Drinking: No	4,880 (69.1)	3,684 (71.00)	1,196 (63.85)	33.00	**<0.001**
Yes	2,182 (30.9)	1,505 (29.00)	677 (36.15)		
Roommates sleep late: No	3,994 (56.56)	3,094 (59.63)	900 (48.05)	75.00	**<0.001**
Yes	3,068 (43.44)	2095 (40.37)	973 (51.95)		
Noisy dormitory: No	5,070 (71.79)	3,923 (75.60)	1,147 (61.24)	140.00	**<0.001**
Yes	1992 (28.21)	1,266 (24.40)	726 (38.76)		
Dormitory light: Not bright	5,043 (71.41)	3,773 (72.71)	1,270 (67.81)	16.00	**<0.001**
Bright	2019 (28.59)	1,416 (27.29)	603 (32.19)		
Dormitory hygiene: Good	2,257 (31.96)	1,561 (30.08)	696 (37.16)	31.00	**<0.001**
Poor	4,805 (68.04)	3,628 (69.92)	1,177 (62.84)		
Romantic relationship status: Never	3,415 (48.36)	2,550 (49.14)	865 (46.18)	6.90	**0.03**
Ever	2,295 (32.50)	1,679 (32.36)	616 (32.89)		
Being in love	1,352 (19.14)	960 (18.50)	392 (20.93)		
Psychological counseling: No	6,261 (88.66)	4,737 (91.29)	1,524 (81.37)	134.00	**<0.001**
Yes	801 (11.34)	452 (8.71)	349 (18.63)		
Grade^∗^: First year	4,530 (64.15)	3,455 (66.58)	1,075 (57.39)	52.00	**<0.001**
Second year	2,239 (31.70)	1,529 (29.47)	710 (37.91)		
Third year	184 (2.61)	133 (2.56)	51 (2.72)		
Fourth year or more	109 (1.54)	72 (1.39)	37 (1.98)		
Father’s education level					
Middle school or less	4,895 (69.31)	3,530 (68.03)	1,365 (72.88)	15.00	**<0.001**
Junior college education	1,147 (16.24)	877 (16.90)	270 (14.42)		
Undergraduate or more	1,020 (14.44)	782 (15.07)	238 (12.71)		
Mother’s education level					
Middle school or less	5,236 (74.14)	3,822 (73.66)	1,414 (75.49)	2.50	0.3
Junior college education	1,039 (14.71)	781 (15.05)	258 (13.77)		
Undergraduate or more	787 (11.14)	586 (11.29)	201 (10.73)		
Family economic level: Good	606 (8.58)	474 (9.13)	132 (7.05)	45.00	**<0.001**
Medium	5,524 (78.22)	4,111 (79.23)	1,413 (75.44)		
Poor	932 (13.20)	604 (11.64)	328 (17.51)		
Monthly living expense (CNY)					
<1,000	852 (12.06)	572 (11.02)	280 (14.95)	21.00	**<0.001**
1,000-2,000	5,222 (73.95)	3,895 (75.06)	1,327 (70.85)		
≥2,000	988 (13.99)	722 (13.91)	266 (14.2)		
Physical exercise: ≤1 per month	1,040 (14.73)	685 (13.20)	355 (18.95)	36.00	**<0.001**
1–3 times per week	4,925 (69.74)	3,680 (70.92)	1,245 (66.47)		
4–7 times per week	1,097 (15.53)	824 (15.88)	273 (14.58)		
Academic pressure: No	304 (4.30)	232 (4.47)	72 (3.84)	120.00	**<0.001**
Normal	5,124 (72.56)	3,928 (75.70)	1,196 (63.85)		
Great	1,634 (23.14)	1,029 (19.83)	605 (32.30)		
Employment pressure: No	1,213 (17.18)	976 (18.81)	237 (12.65)	142.00	**<0.001**
Normal	3,555 (50.34)	2,730 (52.61)	825 (44.05)		
Great	2,294 (32.48)	1,483 (28.58)	811 (43.30)		
Interpersonal relations: Harmonious	4,856 (68.76)	3,755 (72.36)	1,101 (58.78)	124.00	**<0.001**
Ordinary	2,102 (29.76)	1,377 (26.54)	725 (38.71)		
Poor	104 (1.47)	57 (1.10)	47 (2.51)		
Physical health status: Good	3,056 (43.27)	2,490 (47.99)	566 (30.22)	248.00	**<0.001**
Ordinary	3,679 (52.10)	2,543 (49.01)	1,136 (60.65)		
Poor	327 (4.63)	156 (3.01)	171 (9.13)		
Mental health status: Good	3,501 (49.58)	2,925 (56.37)	576 (30.75)	436.00	**<0.001**
Ordinary	3,165 (44.82)	2084 (40.16)	1,081 (57.71)		
Poor	396 (5.61)	180 (3.47)	216 (11.53)		

BMI represents body mass index. ∗ Grade is the term used for undergraduate student years in China. The symbol # denotes that the groups satisfy homoscedasticity and others do not. P values that less than 0.05 were are given in bold.

The PSQI with 19 self-rated items (only the first 18 items contributing to the score) assesses sleep quality over the past month across seven components: subjective sleep quality, sleep latency, sleep duration, habitual sleep efficiency, sleep disturbances, use of sleeping medication, and daytime dysfunction. Each component is scored from 0 to 3, resulting in a total PSQI score ranging from 0 to 21. Higher score indicates poorer sleep quality, with the score above 7 signifying poor sleep quality and the score not greater than 7 indicating good sleep quality. The PSQI score demonstrated good internal consistency in this study since the Cronbach’s *α* was 0.87.

The MPAI with 17 items measures four dimensions: inability to control craving, withdrawal and escape, anxiety and feeling lost, and productivity loss. Items are rated on a 5-point Likert scale (from 1 = not at all to 5 = always), with higher total scores indicating more severe addiction. The scale showed excellent reliability in the present study since the Cronbach’s *α* was 0.93.

Psychological resilience among undergraduates was measured using the CD-RISC, which assesses participants’ feelings over the past month. This 25-item scale uses a 5-point Likert scale (0–4) for each item, ranging from “not true at all” to “true almost all the time.” Total scores range from 0 to 100, with higher scores indicating greater resilience. The CD-RISC demonstrated excellent internal consistency in this study since the Cronbach’s α was 0.969.

### Methodological rationale for model selection

For traditional questionnaire-based data collection, there often exists high multicollinearity and strong correlations between various subsections of the instrument and the overall total score. When examining associations between such questionnaire-derived factors and a given outcome of interest, classical linear or logistic regression is often unsuitable due to the multicollinearity. Although ridge and Lasso regression can mitigate multicollinearity through coefficient shrinkage, these methods distort the original variable space, thereby obscuring the independent contributions of collinear yet conceptually distinct factors. Furthermore, all these linear models assume additive, linear relationships and cannot capture potential nonlinear effects or complex interactions among factors. To address these limitations while maintaining analytical rigor and gaining a more holistic understanding, we developed a complementary dual-model framework combining PLSR and XGBoost. The core rationale for adopting a complementary framework of PLSR and XGBoost lies in their distinct roles: PLSR aims to quantify the strength of linear associations between factors and quantitative PSQI scores while providing interpretable coefficient estimates; XGBoost focuses on identifying nonlinear relationships between factors and the binary classification of sleep quality. Therefore, the differences in their results are not contradictory but rather reveal the complexity of sleep-influencing factors from different dimensions.

### Partial least squares regression (PLSR) model

Multicollinearity occurs when independent variables are highly correlated, leading to unreliable and unstable estimates of regression coefficients. PLSR offers an effective solution of multicollinearity among independent variables (Zheng et al., 2024). It overcomes multicollinearity by extracting orthogonal latent variables that maximize covariance with the outcome variable, thereby projecting high-dimensional collinear data into a lower-dimensional feature space. This approach reduces variable dimensionality while constructing a regression model that maximizes the explained variance of the outcome by the latent components. Finally, the model relationships are mapped back to the original variable space via weight matrices, yielding stable and interpretable coefficient estimates under conditions of multicollinearity. The PLSR model was implemented with the following specifications: the PSQI scores were set as the dependent variable and other variables were all set as the independent variables; continuous independent variables were centered and scaled to unit variance; latent components were selected using 10-fold cross-validation by minimizing the root mean squared error; the partial least squares algorithm was conducted by incorporating jackknife variance estimation for coefficient stability assessment. The PLSR model was developed and evaluated using the ‘pls’ package in R.

### Extreme gradient boosting (XGBoost) model

XGBoost achieves efficient and stable variable selection through its embedded mechanism. It quantifies each variable’s contribution to loss reduction via gain while its built-in L1/L2 regularization automatically suppresses uninformative splits, thereby enabling embedded selection. For a set of highly correlated variables, the tree structure only needs to select one of them to substantially reduce the loss; the remaining variables are not split further due to insufficient gain, avoiding coefficient inflation or deletion of the entire group due to multicollinearity. Moreover, through its multi-segment splitting, XGBoost inherently captures nonlinear patterns such as U-shaped or threshold effects, allowing important variables to automatically stand out in the gain-based ranking. In the present study, we selected the XGBoost model, which has demonstrated superior performance across numerous studies in the machine learning field, to capture nonlinear influencing factors. Furthermore, SHapley Additive exPlanations (SHAP) were employed to provide explanations for the model. PSQI scores were dichotomized as good sleep quality with PSQI ≤ 7 and poor sleep quality with PSQI > 7, which served as the dependent variable. To ensure the robustness and generalizability of our findings, we adopted a rigorous validation protocol. An 8:2 stratified random split was used to allocate 80% of the dataset as a training set, which was used for hyperparameter tuning and model training. The remaining 20% was used for performance validation. SHAP analysis was implemented in the full dataset by XGBoost with the optimal parameters. This separation ensured that the model’s performance was fairly assessed on unseen data. Categorical variables underwent one-hot encoding for model compatibility. To ensure model robustness and mitigate overfitting risks, multiple safeguards were implemented during the XGBoost modeling process. First, structural constraints such as tree depth limits, subsampling, and column subsampling fundamentally control model complexity. Second, hyperparameter tuning via 10-fold cross-validation on the training set were determined by random research method when the parameter combination maximizing the area under the receiver operating characteristic curve (AUC). The final model’s performance was then evaluated on the held-out test set by the metrics of accuracy, AUC, precision, recall and F1. To assess potential bias from gender imbalance, we performed gender-stratified analyses using DeLong’s test to compare the AUC of male-only and female-only XGBoost models. The XGBoost was constructed using ‘mlr’ package in R with poor sleep quality designated as the positive class.

### Statistical analyses

All statistical analyses were conducted using R version 4.3.0. Continuous variables were presented as mean ± standard deviation (SD). Group comparisons were performed using Student’s *t*-test for two groups with equal variances or Welch’s *t*-test for unequal variances. The variance homogeneity was assessed by Levene’s test. Categorical and ordinal variables were described by frequencies (percentages) and compared using $\chi^{2}$ test. The two-side significance threshold was set at 0.05.

## Results

### Basic characteristics

This study utilized a questionnaire with a Cronbach’s *α* coefficient of 0.91, indicating high internal consistency. A total of 7,062 undergraduates were included, comprising 2,086 males (29.54%) and 4,976 females (70.46%). Based on PSQI scores, 1,873 students (26.52%) were classified as poor sleep quality (PSQI>7), while 5,189 students (73.48%) had good sleep quality (PSQI≤7). The basic characteristics of the samples and comparisons between the two sleep quality groups were presented in Table 1. The poor sleep quality group had a significantly higher mean age (*p* < 0.001), and students from rural areas (*p* = 0.020) and only-child households (*p* = 0.001) exhibited a higher prevalence of poor sleep quality. Analysis of family background revealed that students from medium economic level families (*p* < 0.001) and those with monthly living expenses of 1,000–2,000 CNY (*p* < 0.001) had a higher incidence of poor sleep quality. Regarding lifestyle habits, students who smoked, consumed alcohol, or engaged in infrequent physical exercise (≤1 time per month) were more likely to experience sleep problems (all *p* < 0.001). Psychological assessments showed that the poor sleep quality group had significantly lower psychological resilience (CD-RISC total score: 81.40 ± 16.89 vs. 87.17 ± 18.38) and significantly higher smartphone addiction (MPAI total score: 49.41 ± 13.45 vs. 43.33 ± 13.22) (all *p* < 0.001). Additionally, this group reported greater academic pressure and employment pressure (all *p* < 0.001). On dormitory environment, students that with late-sleeping roommates, noisy dormitory, and bright dormitory light encountered with poorer sleep quality (all *p* < 0.001). The PSQI score demonstrated that the poor sleep quality group performed significantly worse across all the seven components, such as sleep latency (2.61 ± 1.54 vs. 0.72 ± 0.95), habitual sleep efficiency (2.09 ± 1.38 vs. 0.46 ± 1.08), and daytime dysfunction (1.00 ± 0.71 vs. 0.46 ± 0.56) (all *p* < 0.001). These findings collectively suggest that college students’ sleep quality is influenced by multifaceted factors, necessitating comprehensive interventions targeting individual behaviors, psychological states, and environmental improvements. Additionally, based on gender, we found that although there were differences in various factors, there were no differences in the classification of sleep quality (Supplementary Table S1).

### Results of PLSR

There existed strong correlations between specific independent variables (see Supplementary Figure S1). We also performed the classical linear regression model and calculated the variance inflation factors (VIFs) for each independent variable to quantitatively assess the multicollinearity (see Supplementary Table S2 and Supplementary Figure S2). There were 10 independent variables with VIF values exceeding 5, indicating multicollinearity existed, including height, weight, BMI, CD-RISC, self-improvement, tenacity, MPAI, inability to control craving, withdrawal and escape, and anxiety and feeling lost. Then we perform PLSR to identifying the influencing factors of PSQI scores for undergraduates in Jiangsu, China. The optimal number of components was determined via 10-fold cross-validation, with the final 8 components selected by minimizing the root mean squared error (see Supplementary Figure S3). Subsequently, loading weights and regression coefficients were analyzed. Finally, jackknife testing was performed to assess the statistical significance of regression coefficients. The detailed findings were summarized in Table 2 and Supplementary Table S3.

**Table 2:** Influencing factors for PSQI scores identified by PLSR.

Variables	Estimates	95% CI	*t* values	*P* values
Age	0.120	[0.015, 0.237]	3.436	**0.007**
CD-RISC	−0.017	[−0.023, −0.012]	−5.951	**<0.001**
Self-improvement	−0.023	[−0.065, 0.018]	−1.263	0.238
Tenacity	−0.017	[−0.039, 0.005]	−1.734	0.117
Optimism	−0.027	[−0.054, 0.049]	−0.122	0.907
MPAI	0.043	[0.038, 0.048]	16.176	**<0.001**
Inability to control craving	0.060	[0.039, 0.081]	6.44	**<0.001**
Withdrawal and escape	0.062	[0.026, 0.099]	3.87	**0.003**
Anxiety and feeling lost	0.034	[−0.002, 0.071]	2.10	0.065
Productivity loss	−0.010	[−0.069, 0.048]	−0.41	0.694
Grade				
First year	−0.250	[−0.368, −0.132]	−4.163	**0.002**
Second year	0.227	[0.054, 0.400]	2.574	**0.003**
Third year	−0.018	[−0.137, 0.101]	−0.302	0.770
Fourth year or more	0.041	[−0.123, 0.205]	0.493	0.634
Monthly living expense (CNY)				
≤1,000	0.131	[0.008, 0.254]	2.086	0.067
1,000-2,000	−0.171	[−0.306, −0.036]	−2.476	**0.035**
≥2,000	0.040	[−0.074, 0.154]	0.685	0.511
Employment pressure				
No	−0.255	[−0.346, −0.164]	−5.463	**<0.001**
Normal	0.049	[−0.071, 0.169]	0.802	0.443
Great	0.206	[0.059, 0.352]	2.760	**0.022**
Interpersonal relations				
Harmonious	−0.172	[−0.251, −0.094]	−4.308	**0.002**
Ordinary	0.029	[−0.099, 0.156]	0.443	0.669
Poor	0.144	[−0.015, 0.293]	2.189	0.056
Physical health status				
Good	−0.595	[−0.781, −0.409]	−6.278	**<0.001**
Ordinary	−0.110	[−0.256, 0.036]	−1.483	0.172
Bad	0.706	[0.454, 0.958]	5.465	**<0.001**
Smoking	0.496	[0.276, 0.716]	4.416	**0.002**
Drinking	0.594	[0.420, 0.768]	6.706	**<0.001**
Mental health status				
Good	−0.853	[−1.079, −0.627]	−7.397	**<0.001**
Ordinary	0.018	[−0.070, 0.106]	0.397	0.701
Bad	0.835	[0.598, 1.072]	6.916	**<0.001**
Noisy dormitory	0.627	[0.420, 0.835]	5.932	**<0.001**
Received psychological counseling	0.746	[0.568, 0.925]	8.216	**<0.001**

The dimensions of CD-RISC and MPAI were included in PLSR model when CD-RISC and MPAI were removed, respectively, with other independent variables retaining. P values that less than 0.05 were given in bold.

Age was positively correlated with PSQI (*β* = 0.120, 95%CI: 0.015 to 0.237, *p* = 0.007), suggesting that older students tend to have poorer sleep quality. Grade level was also associated with sleep quality, with first-year students having better sleep quality (*β* = −0.250, 95%CI: −0.368 to −0.132, *p* = 0.002) and second-year students having poorer sleep quality (*β* = 0.227, 95%CI: 0.054 to 0.400, *p* = 0.003). Students with a moderate monthly living expense (1,000–2,000 CNY) had better sleep quality (*β* = −0.171, 95%CI: −0.306 to −0.036, *p* = 0.035). In terms of lifestyle factors, smoking (*β* = 0.496, 95%CI: 0.276 to 0.716, *p* = 0.002) and drinking (*β* = 0.594, 95%CI: 0.420 to 0.768, *p* < 0.001) were positively associated with PSQI. Additionally, MPAI, including its two dimensions, i.e., inability to control craving, and withdrawal and escape, were positively correlated with PSQI (*β* = 0.060, 95%CI: 0.039 to 0.081, *p* < 0.001; *β* = 0.062, 95%CI: 0.026, 0.099, *p* = 0.003), indicating that students with higher mobile phone dependency had poorer sleep quality. Physical health status was significantly associated with sleep quality, with good physical health status being associated with better sleep quality (*β* = −0.595, 95%CI: −0.781 to −0.409, *p* < 0.001) and poor physical health status being associated with poorer sleep quality (*β* = 0.706, 95%CI: 0.454 to 0.958, *p* < 0.001).

In terms of psychological factors, CD-RISC scores were negatively correlated with PSQI scores (*β* = −0.017, 95%CI: −0.023 to −0.012, *p* < 0.001), suggesting that higher psychological resilience was associated with better sleep quality. However, all the three dimensions of CD-RISC were not significantly associated with PSQI. Students who received psychological counseling had poorer sleep quality (*β* = 0.746, 95%CI: 0.568 to 0.925, *p* < 0.001). The absence of employment pressure and harmonious interpersonal relationships were associated with better sleep quality (*β* = −0.255, 95%CI: −0.346 to −0.164, *p <* 0.001; *β* = −0.172, 95%CI: −0.251 to −0.094, *p =* 0.002). Overall, students with bad mental health status had poorer sleep quality (*β* = 0.835, 95%CI: 0.598 to 1.072, *p* < 0.001), while those with good mental health status had better sleep quality (*β* = −0.853, 95%CI: −1.079 to −0.627, *p* < 0.001). Moreover, a noisy dormitory environment was associated with poorer sleep quality (*β* = 0.627, 95%CI: 0.420 to 0.835, *p* < 0.001). Other factors with no significant associations were also included in Supplementary Table S3.

### Results of XGBoost

**Figure 1:**
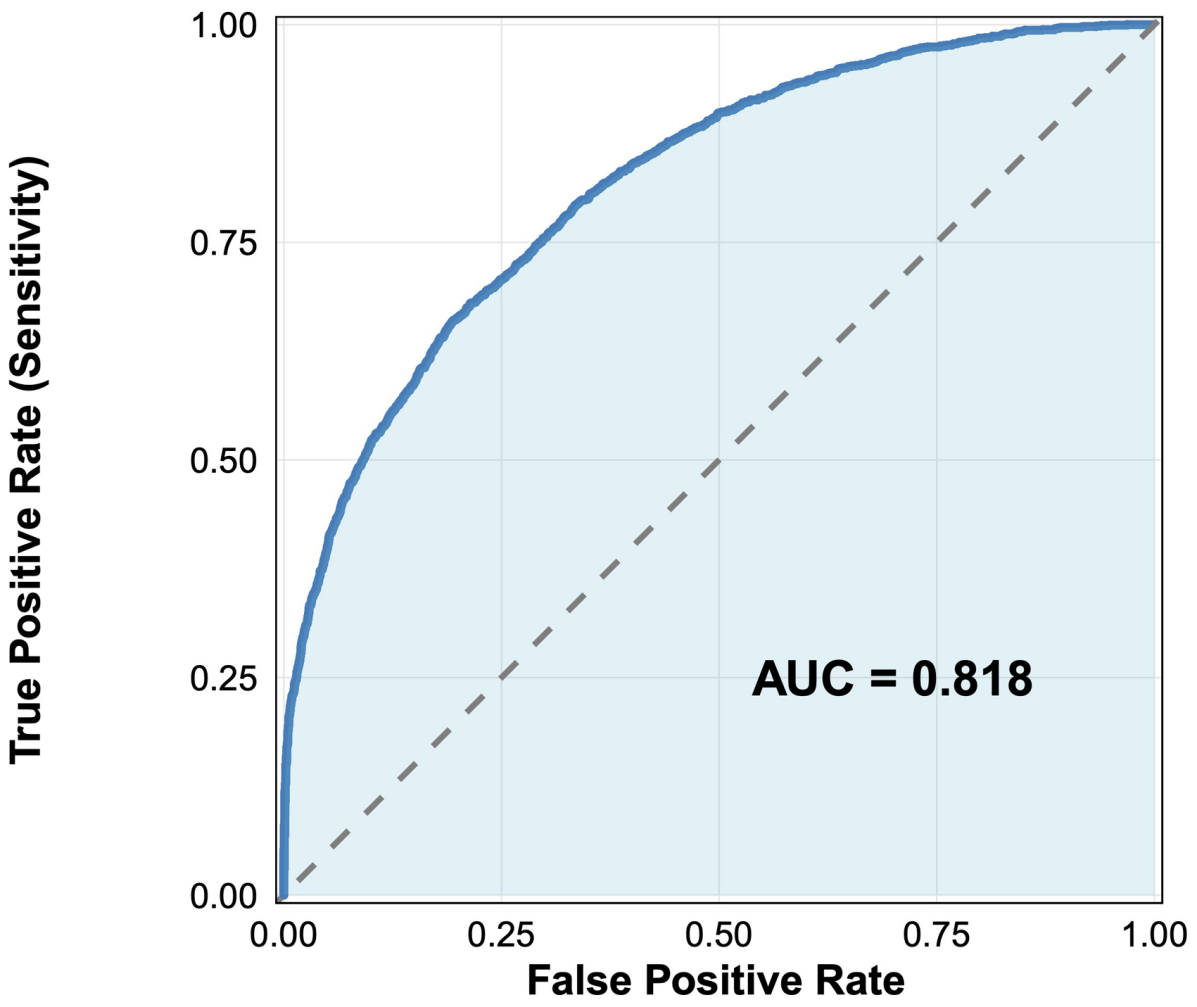
ROC analysis for discriminating between good and poor sleep quality using XGBoost.

The XGBoost classification model was trained on the dataset using the random research method and the constraints of hyperparameters were listed in Supplementary Table S4. There was no significant overfitting or underfitting for XGBoost since the performance metrics were close in the training and test sets, with accuracy achieving at 0.7461 and 0.7321, AUC achieving at 0.7185 and 0.7063, respectively (see Supplementary Table S5). The value of accuracy in the full dataset were 0.7984. And the ROC analysis of the XGBoost model demonstrated an AUC value of 0.818 for discriminating between good and poor sleep quality (Figure 1).

**Figure 2:**
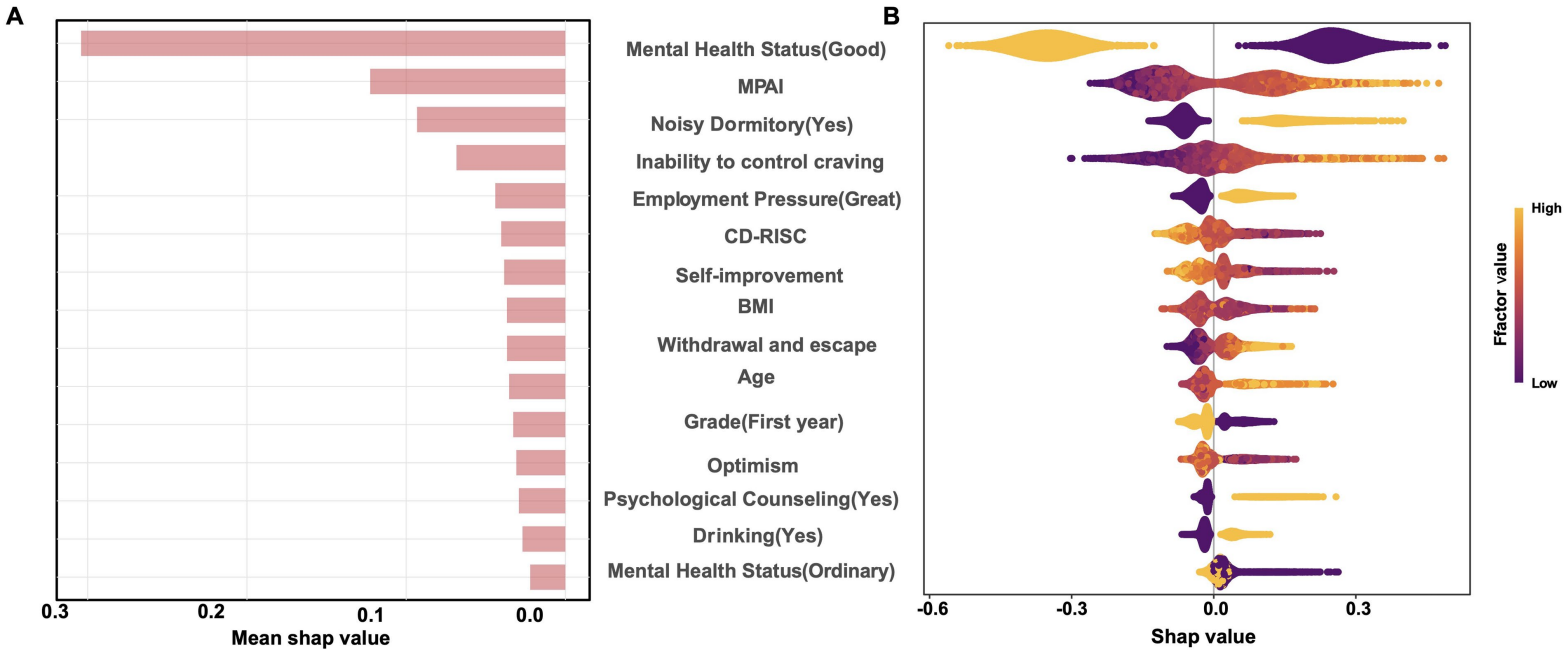
SHAP analysis for XGBoost. **(A)** Shows the factor importance order and **(B)** shows the SHAP value distributions across factors.

The SHAP analyses for XGBoost were shown in Figure 2, including the factor importance order (Figure 2A) and the bee swarm plot (Figure 2B). SHAP-based factor importance analysis revealed that mental health status was the most influential factor of sleep quality, and good mental health status demonstrated positive effect on good sleep quality, while ordinary mental health status ranked last. MPAI also demonstrated high importance in the model, with higher scores being associated with poor sleep quality. Noisy dormitory environment ranked third, followed by greater perceived inability to control smartphone craving, and higher employment pressure, all of which had an impact on poor sleep quality. Moderate influences on poor sleep quality were observed for lower CD-RISC score, lower score of the self-improvement dimension of CD-RISC, BMI, higher score of withdrawal and escape dimension of MPAI, older age, being not a first-year student, lower score of optimism dimension of CD-RISC, having received psychological counseling, and drinking. Notably, although PLSR yielded a non-significant linear coefficient for BMI (*β* = 0.059, 95%CI: −0.04 to 0.168, *p* = 0.729, see Supplementary Table S3), XGBoost ranked BMI among the top factors. Restricted cubic spline analysis confirmed a significant U-shaped relationship between BMI and the risk of poor sleep quality (*p* = 0.012), demonstrating that the tree-based algorithm captured the non-linear pattern missed by the purely linear PLSR model (Supplementary Figure S4). Furthermore, the marginal increase in the PSQI score estimated by XGBoost showed an inflection point at an MPAI score of approximately 49. Thus, an MPAI ≥49 can be tentatively proposed as the high-risk cut-off for campus-based sleep interventions.

### Gender-stratified analysis and model robustness

Given the gender imbalance in our sample (70.46% female), we conducted additional analyses to assess potential bias. DeLong’s test revealed no statistically significant difference in model performance between genders when trained and tested on gender-specific data (male AUC = 0.723, female AUC = 0.699, *p* = 0.105). Gender-stratified SHAP analysis (Supplementary Figures S5, S6) revealed that the top factors were largely consistent across genders: good mental health status, higher CD-RISC scores were positively associated with good sleep quality, while higher MPAI scores and noisy dormitory environments were positively associated with poor sleep quality. However, the magnitude of SHA*p* values for some factors (MPAI had a slightly larger impact in the female model) differed, suggesting nuanced gender-specific patterns. The overall consistency in key factors supported the robustness of our primary conclusions despite the sample imbalance.

## Discussion

### Comparison of PLSR and XGBoost results

Through comparative analysis of the PLSR model and the XGBoost model, we identified both similarities and differences in the factors associated with sleep quality. Both methods consistently identified mental health status as the most influential factor. Common psychological factors identified by both models also included CD-RISC, employment pressure and psychological counseling. Lifestyle factors such as drinking and mobile phone addiction (MPAI) were identified in both models, along with the importance of a noisy dormitory environment. Although the associations of age and grade level were relatively weak, both models were able to identify these factors. These findings suggest that interventions targeting poor sleep quality in college students should not only focus on the most critical psychological factors but also consider their lifestyle and diverse background contexts.

However, PLSR identified several notable demographic and lifestyle factors (monthly living expenses, smoking, and physical health status) as well as interpersonal factors (interpersonal relationships). In contrast, XGBoost identified BMI, which was not noted by PLSR. This divergence is methodologically informative: PLSR with PSQI scores being the outcome exceled at estimating the linear contribution of each variable while managing collinearity, making it sensitive to demographic and straightforward behavioral factors. XGBoost with a binary outcome automatically detected and leveraged nonlinear relationships and interactions through its tree-based structure. These differences highlighted the complementary nature of linear (PLSR) and nonlinear (XGBoost) models in analyzing the complex factors of sleep quality. The identification of BMI by XGBoost but not by PLSR was a prime example. The linear PLSR model found no significant straight-line relationship, but XGBoost revealed a significant U-shaped association (lowest risk at BMI = 21 kg/m^2^, with risk increasing at both lower and higher values), which was confirmed by restricted cubic spline analysis. This highlights how linear models can miss important but nonlinear effects, underscoring the value of our complementary approach.

The non-significant difference in AUC between gender-specific models suggested that the overall model’s performance was not substantially biased by the gender imbalance in our sample. This finding, coupled with the consistency in top factors identified by SHAP analysis across genders, supported the robustness of our primary conclusions. The consistent identification of factors across gender-stratified models suggested that core sleep-related factors exhibited similarly patterns of associations for both sexes. This finding mitigated concerns regarding potential bias from gender imbalance in our sample.

### Psychological and physiological factors of sleep quality

Physical and mental health status, and psychological resilience have all been identified as closely associated with sleep quality (Tsai et al., 2022; Cai et al., 2023; Damgaard et al., 2024). Deteriorated physical condition, such as chronic pain, can directly disrupt normal sleep processes (Tsai et al., 2022). Additionally, existing research demonstrated that prolonged sleep deprivation adversely affected hormonal balance and immune function, leading to metabolic dysregulation and chronic illnesses, thereby establishing a vicious cycle between these factors (Mukherjee et al., 2024). In terms of mental health, negative emotions like anxiety and depression were significant contributors to the onset of insomnia (Damgaard et al., 2024). The present study utilized quantitative changes to elucidate the relationship between psychological status and PSQI scores, revealing that poorer psychological states were associated with poor sleep quality, while better psychological states corresponded to improved sleep quality. These findings aligned with previous research, which indicated that negative emotional states activated the sympathetic nervous system, thereby interfering with the regular sleep–wake cycle (Delannoy et al., 2015; Kim et al., 2024). Similarly, psychological resilience appeared to function as a protective factor; individuals with high resilience were better equipped to manage stress and regulate emotions, thereby diminishing the detrimental impact of stressors on sleep (Shi et al., 2022; Xie et al., 2023). Additionally, we identified the correlation between self-rated physical health status and PSQI scores, although further evidence is needed to elucidate the underlying associations. Additionally, undergoing psychological therapy and having occupational stress were both associated with higher PSQI scores. In the quantitative analysis using PLSR, the absence of employment stress showed a negative association with PSQI, while high employment stress exhibited a positive association. Consequently, enhancing the physical health status of undergraduates, refining the mental health support frameworks, and systematically fostering their resilience and emotional regulation capabilities may significantly contribute to the improvement of their sleep quality.

### Behavioral and digital lifestyle impacts on sleep quality

The influence of mobile phone addiction on sleep was particularly pronounced, aligning with a substantial body of recent research conducted both domestically and internationally (Li et al., 2020b). Factors such as blue light emission from mobile screens, information overload, and emotional fluctuations resulting from social media usage disrupted normal sleep rhythms (LeBourgeois et al., 2017; Silvani et al., 2022; Yu et al., 2024). Empirical evidence demonstrated that blue light emitted by digital devices, including smartphones and tablets, suppressed the synthesis of melatonin, a hormone indispensable for regulating the sleep–wake cycle; this suppression may consequently lead to delayed sleep onset and compromised sleep quality (Cajochen et al., 2011). Furthermore, the continuous influx of information originating from digital media sources generated psychological stimulation, which may consequently postpone the initiation of sleep and subsequently reduced the overall duration of sleep (LeBourgeois et al., 2017). Emotional engagement and negative interactions on social media platforms can induce stress and anxiety, leading to sleep disturbances, while excessive use exacerbates emotional disorders such as depression and anxiety, further compromising both sleep quality and duration (Dresp-Langley and Hutt, 2022; Yu et al., 2024). Consequently, persistent use of mobile phones before bedtime leads to difficulties in falling asleep, reduced sleep duration, and decreased sleep quality.

Furthermore, the detrimental effects of drinking and smoking on sleep have been extensively validated. Excessive alcohol consumption was correlated with compromised sleep quality, presenting as an escalation in insomnia symptoms, reduced sleep duration, and disruptions in circadian rhythms (He et al., 2019). Alcohol modulated the brain’s adenosine pathways, which were essential for regulating sleep drive and maintaining sleep homeostasis. This interference may result in increased wakefulness during sleep periods and a consequent reduction in overall sleep quantity (Sharma et al., 2018). Smoking reduced the duration of both slow-wave and rapid eye movement sleep stages, thereby disrupting the structural framework of sleep architecture (Grigoriou et al., 2024). Additionally, nicotine, a stimulant present in tobacco, heightened wakefulness and induced sleep fragmentation, further impairing established sleep patterns (Jang et al., 2023). These findings highlighted the critical importance of advocating for healthy lifestyles, minimizing mobile phone dependence, and limiting the intake of tobacco and alcohol as essential strategies for enhancing sleep quality among undergraduates.

### Effect environmental conditions and socio-psychological factors on sleep quality

The study identified complex associations between socio-environmental factors and sleep outcomes. Both PLSR and XGBoost analyses consistently confirmed the negative association of noisy dormitory environments with sleep quality. Specifically, dormitory noise disrupted sleep architecture, increased the number of awakenings, and decreased sleep efficiency (Nakagawa, 1987; Yamagami et al., 2023).

Harmonious interpersonal relation demonstrated a negative association with PSQI in the PLSR analysis, indicating that it related with better sleep quality, which was consistent with previous research findings (van Schalkwijk et al., 2015). Although neither model identified a significant correlation between higher grade levels and sleep quality, both explained that first-year students had better sleep quality. Notably, in terms of linear relationships, PLSR detected that second-year students had poorer sleep quality, further highlighting the rich interpretability of linear and nonlinear models.

In the PLSR model, we also found an association between monthly living expenses and PSQI scores, which has drawn our attention. We will continue to collect data to validate the underlying relationship between financial capacity and sleep quality.

### Limitations

This study built upon the same database of college student sleep quality established in our previous work (Hu et al., 2024). Methodologically, it represented a significant advancement. Unlike the traditional regression methods used in earlier studies, which required variable selection to address multicollinearity, this study adopted a “PLSR-XGBoost” hybrid framework. Although this study identified multiple factors influencing the sleep quality of undergraduates in Jiangsu Province, certain limitations remain. Primarily, as a cross-sectional investigation, it was incapable of establishing causal relationships between variables. And the use of convenience sampling may introduce selection bias as the sample predominantly consists of undergraduates from specific departments or dormitory clusters, potentially overlooking broader student demographics with varying sleep patterns. Furthermore, the research predominantly relied on students’ self-reported data, which may be susceptible to recall bias or social desirability bias. Despite the gender imbalance, our stratified analyses and robustness checks suggested the core findings were not substantially biased. Future studies should adopt longitudinal designs to explore additional potential factors affecting sleep and their interactions comprehensively, while also conducting in-depth analyses of subgroups based on different academic years, majors, and genders.

## Conclusion

This study investigated sleep quality and its influencing factors among college students in Jiangsu Province using an integrated PLSR-XGBoost approach. The key findings revealed that: (1) poor sleep quality affected over 25% of students, with mental health status being the most influential factor; (2) environmental factors (noise dormitory) and lifestyle habits (MPAI, smoking, drinking) significantly impaired sleep quality; (3) factors like BMI exhibited nonlinear (U-shaped) relationships with sleep quality; and (4) a tentative high-risk threshold for smartphone dependence (MPAI ≥49) was identified. These results highlighted two priority areas for intervention: (1) mental health support, especially for students with subclinical psychological distress; (2) environment optimization, including noise reduction in dormitories.

## Data Availability

The raw data supporting the conclusions of this article will be made available by the authors, without undue reservation.

## Footnotes

1
www.wjx.cn
