# Supplementary material for: Identifying influencing factors associated with sleep quality in undergraduates based on partial least squares regression and XGBoost
Source: Front Psychol. 2026 Jan 12;16:1732946. doi: 10.3389/fpsyg.2025.1732946 (PMC12832399; doi:10.3389/fpsyg.2025.1732946)
Supplement: Supplementary file 1 [file Table_1.docx]

**Supplementary materials for**

**Identifying influencing factors associated with sleep quality in undergraduates based on partial least squares regression and XGBoost**

Yuchen Xie^1,✝^, Yuan Chen^2, ✝^, Yaohui Han^2^, Shilei Zhai^2^, Lishun Xiao^2, *^, Dehui Yin^2, *^ and [Yansu Chen](https://pubmed.ncbi.nlm.nih.gov/?term=Chen%20Y%5BAuthor%5D)^2,*^

1 Xuzhou Medical University, Xuzhou, 221004, Jiangsu, China

2 School of Public Health, Xuzhou Medical University, Xuzhou, 221004, Jiangsu, China

^✝^These authors contributed equally to this research.

^*^Corresponding author: Yansu Chen (chenyansu@xzhmu.edu.cn); Lishun Xiao [(xiaolishun@xzhmu.edu.cn) and](mailto:(xiaolishun@xzhmu.edu.cn)and) Dehui Yin (yindh16@xzhmu.edu.cn)

1. Supplementary figures and tables


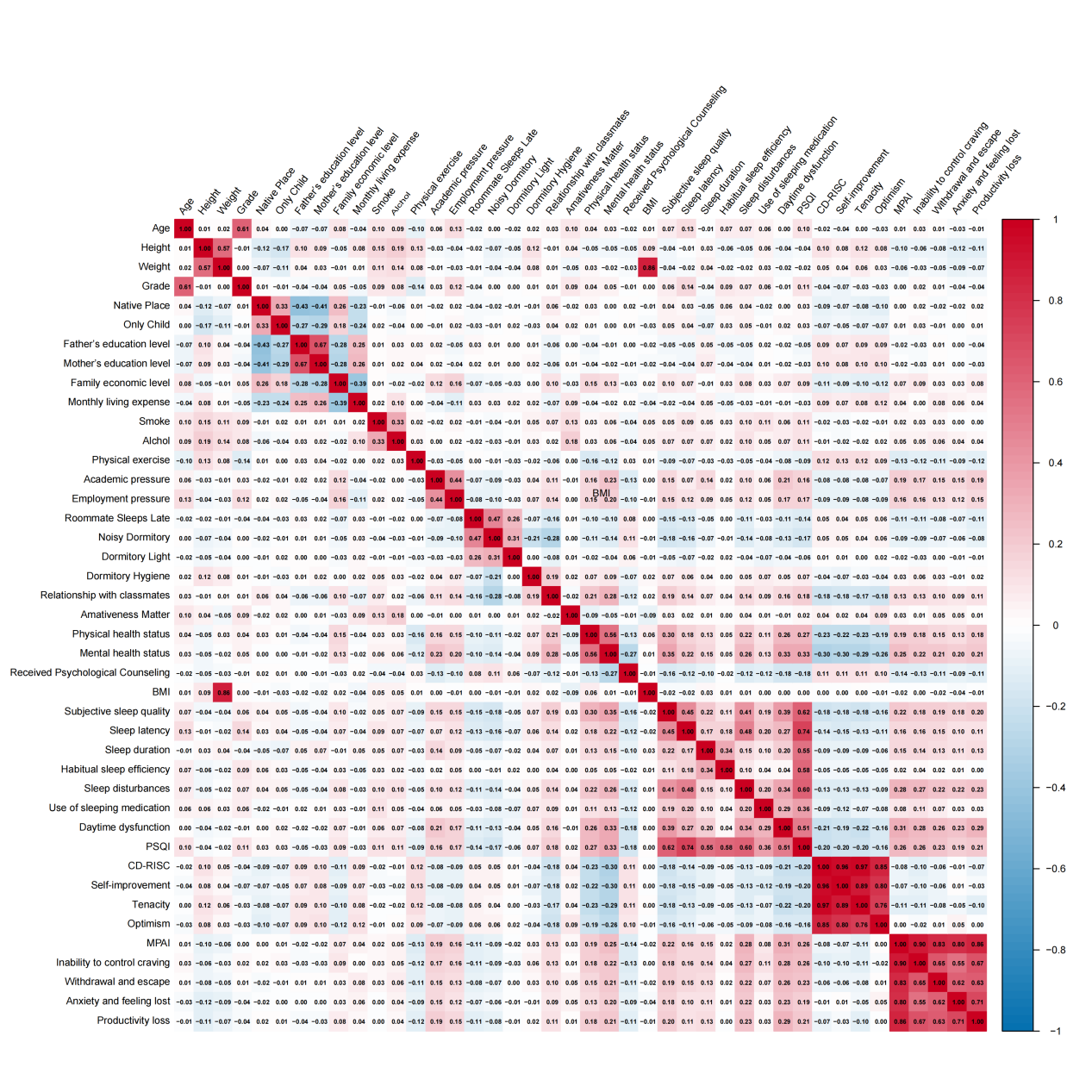


Figure S1 Heatmap of the correlation matrix between independent variables

Table S1 The basic characteristics of undergraduates in Jiangsu, China

| Variables | Total (*n* = 7062) | Female (*n* = 4976) | Male (*n* = 2086) | $t/\chi^{2}$ | *P* |
| --- | --- | --- | --- | --- | --- |
|  |  |  |  |  |  |
| Age (year) | 18.72 ± 0.95 | 18.67 ± 0.92 | 18.82 ± 1.01 | -5.93 | **<0.001^#^** |
| Height (cm) | 167.54 ± 8.40 | 163.63 ± 5.64 | 176.87 ± 6.29 | -83.15 | **<0.001^#^** |
| Weight (kg) | 60.53 ± 12.66 | 56.41 ± 10.27 | 70.36 ± 12.42 | -45.22 | **<0.001^#^** |
| BMI (kg/m^2^) | 21.48 ± 3.75 | 21.07 ± 3.74 | 22.45 ± 3.58 | -14.64 | **<0.001^#^** |
| PSQI | 5.64 ± 3.59 | 5.70 ± 3.50 | 5.50 ± 3.79 | 2.03 | **0.043** |
| Sleep latency | 1.22 ± 1.41 | 1.23 ± 1.40 | 1.21 ± 1.42 | 0.52 | 0.600 |
| Use of sleeping medication | 0.11 ± 0.43 | 0.09 ± 0.39 | 0.16 ± 0.52 | -5.84 | **<0.001** |
| Daytime dysfunction | 0.60 ± 0.65 | 0.62 ± 0.64 | 0.57 ± 0.68 | 2.82 | **0.005** |
| Habitual sleep efficiency | 0.89 ± 1.37 | 0.93 ± 1.39 | 0.80 ± 1.32 | 3.89 | **<0.001** |
| Subjective sleep quality | 0.91 ± 0.63 | 0.92 ± 0.61 | 0.87 ± 0.68 | 3.22 | **0.001** |
| Sleep disturbances | 0.97 ± 0.65 | 0.99 ± 0.63 | 0.91 ± 0.71 | 4.74 | **<0.001** |
| Sleep duration | 0.94 ± 0.88 | 0.92 ± 0.87 | 0.99 ± 0.89 | -3.28 | **0.001** |
| CD-RISC | 85.64 ± 18.18 | 84.74 ± 16.82 | 87.79 ± 20.91 | -5.90 | **<0.001^#^** |
| Self-improvement | 28.58 ± 6.04 | 28.37 ± 5.61 | 29.09 ± 6.96 | -4.19 | **<0.001** |
| Tenacity | 43.77 ± 10.00 | 43.19 ± 9.35 | 45.15 ± 11.27 | -6.99 | **<0.001** |
| Optimism | 13.29 ± 3.11 | 13.18 ± 2.92 | 13.56 ± 3.50 | -4.26 | **<0.001** |
| MPAI | 44.94 ± 13.55 | 46.04 ± 12.97 | 42.32 ± 14.53 | 10.14 | **<0.001^#^** |
| Inability to control craving | 19.20 ± 6.11 | 19.48 ± 5.84 | 18.56 ± 6.66 | 5.50 | **<0.001^#^** |
| Withdrawal and escape | 7.84 ± 3.46 | 8.08 ± 3.46 | 7.27 ± 3.39 | 9.05 | **<0.001^#^** |
| Anxiety and feeling lost | 8.97 ± 3.21 | 9.31 ± 3.10 | 8.16 ± 3.32 | 13.47 | **<0.001^#^** |
| Productivity loss | 8.93 ± 3.05 | 9.18 ± 2.94 | 8.33 ± 3.21 | 10.35 | **<0.001^#^** |
| Grade∗ |  |  |  | 92.17 | **<0.001** |
| First year | 4530 (64.15) | 3092 (62.14) | 1438 (68.94) |  |  |
| Second year | 2239 (31.70) | 1721 (34.59) | 518 (24.83) |  |  |
| Third year | 184 (2.61) | 112 (2.25) | 72 (3.45) |  |  |
| Fourth year or more | 109 (1.54) | 51 (1.02) | 58 (2.78) |  |  |
| Native place |  |  |  | 8.13 | **0.004** |
| Rural | 4294 (60.80) | 3079 (61.88) | 1215 (58.25) |  |  |
| Urban | 2768 (39.20) | 1897 (38.12) | 871 (41.75) |  |  |
| Only child |  |  |  | 139.07 | **<0.001** |
| No | 4366 (61.82) | 3296 (66.24) | 1070 (51.29) |  |  |
| Yes | 2696 (38.18) | 1680 (33.76) | 1016 (48.71) |  |  |
| Father education level |  |  |  | 8.01 | **0.018** |
| Junior college education | 1147 (16.24) | 833 (16.74) | 314 (15.05) |  |  |
| Middle school or less | 4895 (69.31) | 3458 (69.49) | 1437 (68.89) |  |  |
| Undergraduate or more | 1020 (14.44) | 685 (13.77) | 335 (16.06) |  |  |
| Mother education level |  |  |  | 5.30 | 0.070 |
| Junior college education | 1039 (14.71) | 759 (15.25) | 280 (13.42) |  |  |
| Middle school or less | 5236 (74.14) | 3680 (73.95) | 1556 (74.59) |  |  |
| Undergraduate or more | 787 (11.14) | 537 (10.79) | 250 (11.98) |  |  |
| Monthly living expense |  |  |  | 18.18 | **<0.001** |
| <1,000 | 852 (12.06) | 630 (12.66) | 222 (10.64) |  |  |
| >2,000 | 988 (13.99) | 738 (14.83) | 250 (11.98) |  |  |
| 1,000-2,000 | 5222 (73.95) | 3608 (72.51) | 1614 (77.37) |  |  |
| Family economic level |  |  |  | 14.27 | **<0.001** |
| Good | 606 (8.58) | 441 (8.86) | 165 (7.91) |  |  |
| Medium | 5524 (78.22) | 3926 (78.90) | 1598 (76.61) |  |  |
| Poor | 932 (13.20) | 609 (12.24) | 323 (15.48) |  |  |
| Smoking, |  |  |  | 197.99 | **<0.001** |
| No | 6724 (95.21) | 4853 (97.53) | 1871 (89.69) |  |  |
| Yes | 338 (4.79) | 123 (2.47) | 215 (10.31) |  |  |
| Drinking |  |  |  | 323.19 | **<0.001** |
| No | 4880 (69.10) | 3757 (75.50) | 1123 (53.84) |  |  |
| Yes | 2182 (30.90) | 1219 (24.50) | 963 (46.16) |  |  |
| Physical exercise |  |  |  | 154.24 | **<0.001** |
| 1-3Times per week | 4925 (69.74) | 3558 (71.50) | 1367 (65.53) |  |  |
| 4-7Time per week | 1097 (15.53) | 608 (12.22) | 489 (23.44) |  |  |
| ≤1 per month | 1040 (14.73) | 810 (16.28) | 230 (11.03) |  |  |
| Academic pressure: |  |  |  | 32.33 | **<0.001** |
| Great | 1634 (23.14) | 1158 (23.27) | 476 (22.82) |  |  |
| No | 304 (4.30) | 170 (3.42) | 134 (6.42) |  |  |
| Normal | 5124 (72.56) | 3648 (73.31) | 1476 (70.76) |  |  |
| Employment pressure |  |  |  | 16.45 | **<0.001** |
| Great | 2294 (32.48) | 1621 (32.58) | 673 (32.26) |  |  |
| No | 1213 (17.18) | 798 (16.04) | 415 (19.89) |  |  |
| Normal | 3555 (50.34) | 2557 (51.39) | 998 (47.84) |  |  |
| Roommate sleeps late |  |  |  | 5.55 | **0.018** |
| No | 3994 (56.56) | 2859 (57.46) | 1135 (54.41) |  |  |
| Yes | 3068 (43.44) | 2117 (42.54) | 951 (45.59) |  |  |
| Noisy dormitory |  |  |  | 37.46 | **<0.001** |
| No | 5070 (71.79) | 3678 (73.91) | 1392 (66.73) |  |  |
| Yes | 1992 (28.21) | 1298 (26.09) | 694 (33.27) |  |  |
| Dormitory light |  |  |  | 54.28 | **<0.001** |
| Bright | 2019 (28.59) | 1295 (26.02) | 724 (34.71) |  |  |
| Not bright | 5043 (71.41) | 3681 (73.98) | 1362 (65.29) |  |  |
| Dormitory hygiene |  |  |  | 98.37 | **<0.001** |
| Good | 2257 (31.96) | 1413 (28.40) | 844 (40.46) |  |  |
| Poor | 4805 (68.04) | 3563 (71.60) | 1242 (59.54) |  |  |
| Interpersonal relations |  |  |  | 30.46 | **<0.001** |
| Harmonious | 4856 (68.76) | 3427 (68.87) | 1429 (68.50) |  |  |
| Ordinary | 2102 (29.76) | 1501 (30.16) | 601 (28.81) |  |  |
| Poor | 104 (1.47) | 48 (0.96) | 56 (2.68) |  |  |
| Romantic relationship status |  |  |  | 22.99 | **<0.001** |
| Being in love | 1352 (19.14) | 977 (19.63) | 375 (17.98) |  |  |
| Ever | 2295 (32.50) | 1531 (30.77) | 764 (36.63) |  |  |
| Never | 3415 (48.36) | 2468 (49.60) | 947 (45.40) |  |  |
| Physical health status |  |  |  | 14.79 | **<0.001** |
| Bad | 327 (4.63) | 212 (4.26) | 115 (5.51) |  |  |
| Good | 3056 (43.27) | 2104 (42.28) | 952 (45.64) |  |  |
| Ordinary | 3679 (52.10) | 2660 (53.46) | 1019 (48.85) |  |  |
| Mental health status |  |  |  | 52.79 | **<0.001** |
| Bad | 396 (5.61) | 259 (5.20) | 137 (6.57) |  |  |
| Good | 3501 (49.58) | 2349 (47.21) | 1152 (55.23) |  |  |
| Ordinary | 3165 (44.82) | 2368 (47.59) | 797 (38.21) |  |  |
| Received psychological counseling |  |  |  | 11.59 | **<0.001** |
| No | 6261 (88.66) | 4453 (89.49) | 1808 (86.67) |  |  |
| Yes | 801 (11.34) | 523 (10.51) | 278 (13.33) |  |  |
| PSQI level |  |  |  | 2.79 | 0.095 |
| Good | 5189 (73.48) | 3628 (72.91) | 1561 (74.83) |  |  |
| Poor | 1873 (26.52) | 1348 (27.09) | 525 (25.17) |  |  |
| Notes: ∗Grade is the term used for undergraduate student years in China. #Denotes that the groups satisfy homoscedasticity and others do not. P values that less than 0.05 are given in bold. | | | | | |

Table S2 Multicollinearity detection for the linear regression model

| Variables | VIF | 95%CI of VIF | Tolerance | 95%CI of Tolerance |
| --- | --- | --- | --- | --- |
| Gender | 2.35 | [ 2.27, 2.44] | 0.42 | [0.41, 0.44] |
| Age | 1.76 | [ 1.70, 1.82] | 0.57 | [0.55, 0.59] |
| Height | 32.66 | [ 31.21, 34.19] | 0.03 | [0.03, 0.03] |
| Weight | 127.10 | [121.37, 133.10] | 0.01 | [0.01, 0.01] |
| BMI | 86.35 | [ 82.47, 90.42] | 0.01 | [0.01, 0.01] |
| CD-RISC | 101.76 | [97.17, 106.56] | 0.01 | [0.01, 0.01] |
| Self-improvement | 23.94 | [ 22.88, 25.05] | 0.04 | [0.04, 0.04] |
| Tenacity | 40.44 | [ 38.63, 42.33] | 0.02 | [0.02, 0.03] |
| Optimism | 35.20 | [33.50, 37.00] | 0.02 | [0.02, 0.03] |
| MPAI | 55.70 | [ 53.21, 58.32] | 0.02 | [0.02, 0.02] |
| Inability to control craving | 17.31 | [ 16.55, 18.11] | 0.06 | [0.06, 0.06] |
| Withdrawal and escape | 6.61 | [ 6.34, 6.91] | 0.15 | [0.14, 0.16] |
| Anxiety and feeling lost | 7.99 | [ 7.65, 8.35] | 0.13 | [0.12, 0.13] |
| Grade | 1.86 | [ 1.80, 1.92] | 0.54 | [0.52, 0.56] |
| Native Place | 1.39 | [ 1.35, 1.43] | 0.72 | [0.70, 0.74] |
| Only Child | 1.24 | [ 1.21, 1.28] | 0.81 | [0.78, 0.83] |
| Father’s education level | 2.33 | [ 2.25, 2.42] | 0.43 | [0.41, 0.45] |
| Mother’s education level | 2.35 | [ 2.27, 2.44] | 0.42 | [0.41, 0.44] |
| Family economic level | 1.50 | [ 1.45, 1.55] | 0.67 | [0.65, 0.69] |
| Monthly living expense (CNY) | 1.45 | [ 1.40, 1.49] | 0.69 | [0.67, 0.71] |
| Smoking | 1.15 | [ 1.12, 1.18] | 0.87 | [0.85, 0.89] |
| Drinking | 1.19 | [ 1.16, 1.23] | 0.84 | [0.82, 0.86] |
| Physical exercise | 1.15 | [ 1.13, 1.19] | 0.87 | [0.84, 0.89] |
| Academic pressure | 1.56 | [ 1.51, 1.61] | 0.64 | [0.62, 0.66] |
| Employment pressure | 1.56 | [ 1.51, 1.61] | 0.64 | [0.62, 0.66] |
| Relationship with classmates | 1.29 | [ 1.25, 1.33] | 0.78 | [0.75, 0.80] |
| Roommates sleep late | 1.34 | [ 1.30, 1.38] | 0.75 | [0.72, 0.77] |
| Noisy dormitory | 1.50 | [ 1.45, 1.54] | 0.67 | [0.65, 0.69] |
| Dormitory light | 1.15 | [ 1.13, 1.19] | 0.87 | [0.84, 0.89] |
| Dormitory hygiene | 1.11 | [ 1.08, 1.14] | 0.90 | [0.88, 0.92] |
| Amativeness Matter | 1.13 | [ 1.10, 1.16] | 0.89 | [0.86, 0.91] |
| Physical health status | 1.77 | [ 1.71, 1.83] | 0.57 | [0.55, 0.58] |
| Mental health status | 2.07 | [ 2.00, 2.15] | 0.48 | [0.47, 0.50] |
| Received psychological counseling | 1.14 | [ 1.11, 1.17] | 0.88 | [0.86, 0.90] |


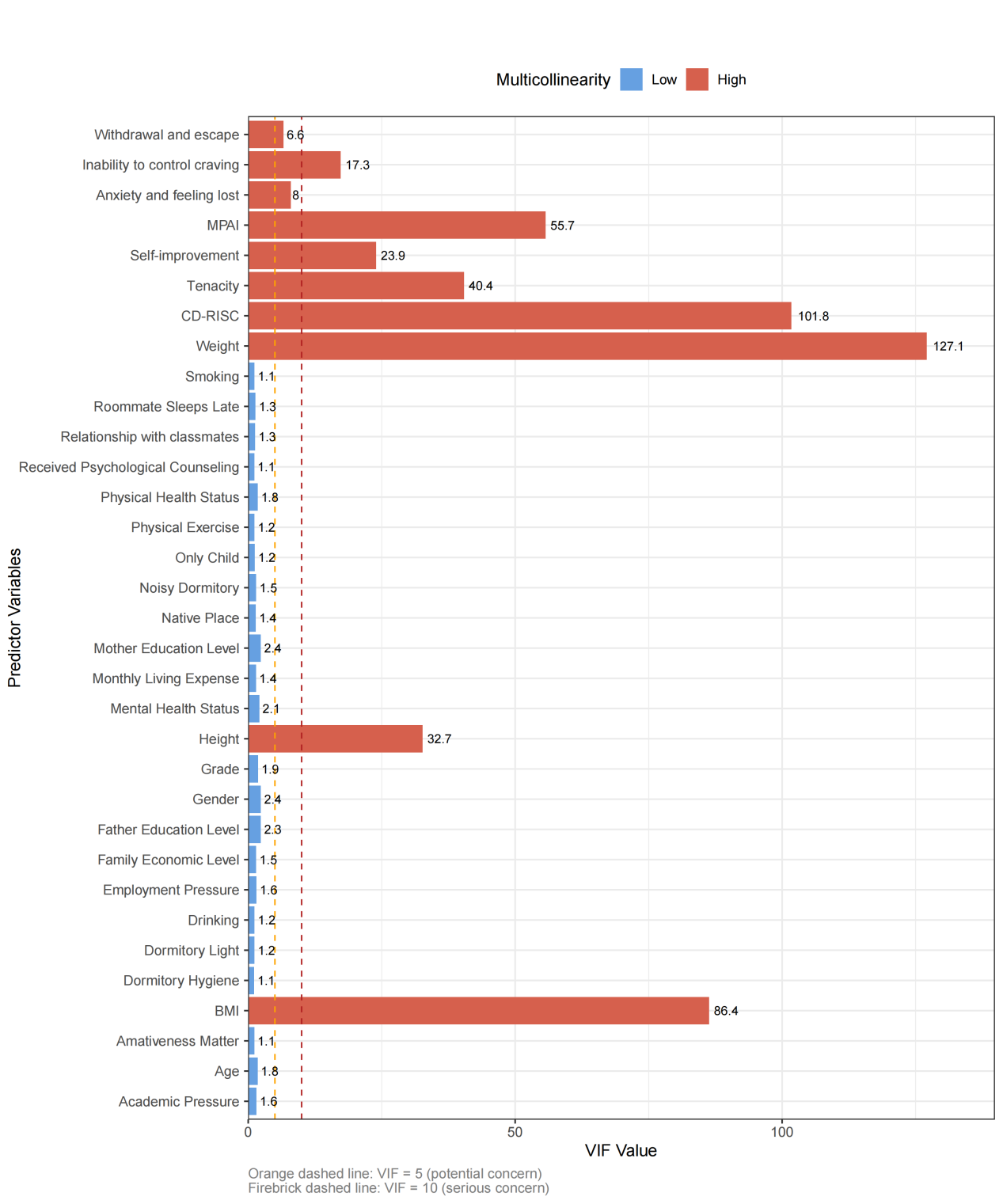


Figure S2 Variance inflation factors (VIF) for independent variables


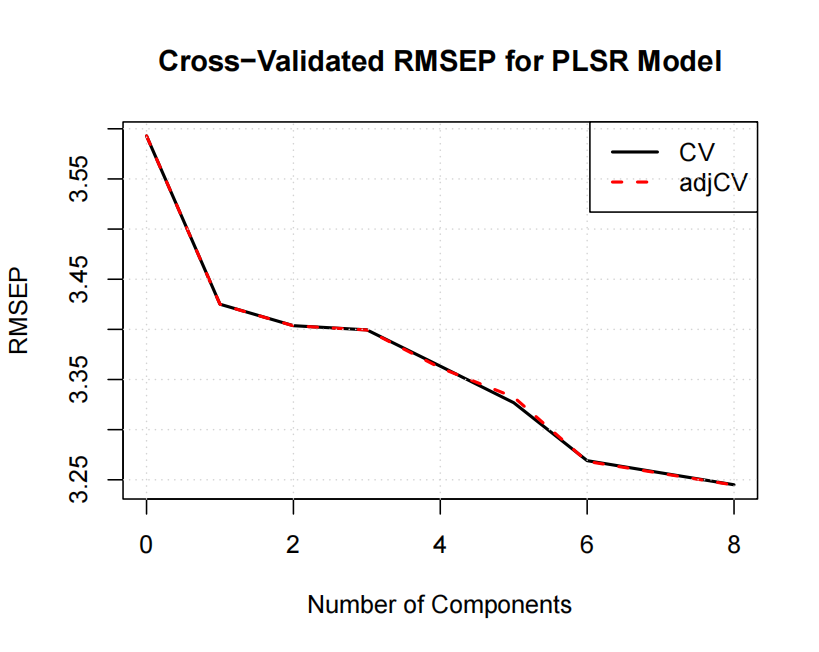


Figure S3 Determination of component numbers for PLSR

Table S3 PLSR results with all the independent variables

| Variables | Estimate | 95% CI | *t* value | *P* values |
| --- | --- | --- | --- | --- |
| Gender | 0.011 | [-0.199, 0.221] | 0.114 | 0.911 |
| Age | 0.120 | [0.015, 0.237] | 3.436 | **0.007** |
| Height | 0.012 | [-0.016, 0.042] | 0.256 | 0.803 |
| Weight | -0.024 | [-0.064, 0.012] | -0.403 | 0.696 |
| BMI | 0.059 | [-0.04, 0.168] | 0.356 | 0.729 |
| CD-RISC | -0.017 | [-0.023, -0.012] | -5.951 | **<0.001** |
| Self-improvement | -0.02 | [-0.06, 0.02] | -0.835 | 0.425 |
| Tenacity | 0.01 | [-0.01, 0.03] | 1.186 | 0.266 |
| Optimism | -0.01 | [-0.06, 0.04] | -0.483 | 0.641 |
| MPAI | 0.043 | [0.038, 0.048] | 16.176 | **<0.001** |
| Inability to control craving | 0.03 | [0.01, 0.05] | 2.943 | **0.016** |
| Withdrawal and escape | -0.02 | [-0.07, 0.03] | -0.642 | 0.537 |
| Anxiety and feeling lost | 0.03 | [0.00, 0.05] | 2.049 | 0.071 |
| Productivity loss | 0.00 | [-0.04, 0.03] | -0.164 | 0.874 |
| Grade |  |  |  |  |
| First year | -0.250 | [-0.368, -0.132] | -4.163 | **0.002** |
| Second year | 0.227 | [0.054, 0.400] | 2.574 | **0.003** |
| Third year | -0.018 | [-0.137, 0.101] | -0.302 | 0.770 |
| Fourth year or more | 0.041 | [-0.123, 0.205] | 0.493 | 0.634 |
| Father’s education level |  |  |  |  |
| Middle school or less | 0.143 | [-0.029, 0.315] | 1.630 | 0.088 |
| Junior college education | -0.073 | [-0.191, 0.045] | -0.974 | 0.355 |
| Undergraduate or more | -0.070 | [-0.307, 0.168] | -0.999 | 0.344 |
| Mother’s education level |  |  |  |  |
| Middle school or less | -0.038 | [-0.117, 0.040] | -0.955 | 0.365 |
| Junior college education | 0.016 | [-0.132, 0.164] | 0.209 | 0.839 |
| Undergraduate or more | 0.022 | [-0.149, 0.194] | 0.256 | 0.803 |
| Family economic level |  |  |  |  |
| Good | 0.022 | [-0.072, 0.116] | 0.461 | 0.656 |
| Medium | -0.062 | [-0.192, 0.068] | -0.936 | 0.374 |
| Poor | 0.040 | [-0.124, 0.204] | 0.479 | 0.644 |
| Monthly living expense (CNY) |  |  |  |  |
| ≤1,000 | 0.131 | [0.008, 0.254] | 2.086 | 0.067 |
| 1,000-2,000 | -0.171 | [-0.306, -0.036] | -2.476 | **0.035** |
| ≥2,000 | 0.040 | [-0.074, 0.154] | 0.685 | 0.511 |
| Physical exercise |  |  |  |  |
| once per month | 0.120 | [-0.040, 0.280] | 1.471 | 0.175 |
| 1-3Times per week | -0.050 | [-0.178, 0.078] | -0.762 | 0.465 |
| 4-7Times per week | -0.077 | [-0.233, 0.093] | -0.842 | 0.422 |
| Academic pressure |  |  |  |  |
| No | -0.082 | [-0.289, 0.125] | -0.776 | 0.458 |
| Normal | -0.100 | [-0.287, 0.088] | -1.039 | 0.326 |
| Great | 0.181 | [-0.010, 0.373] | 1.858 | 0.096 |
| Employment pressure |  |  |  |  |
| No | -0.255 | [-0.346, -0.164] | -5.463 | **<0.001** |
| Normal | 0.049 | [-0.071, 0.169] | 0.802 | 0.443 |
| Great | 0.206 | [0.059, 0.352] | 2.760 | **0.022** |
| Interpersonal relations |  |  |  |  |
| Harmonious | -0.172 | [-0.251, -0.094] | -4.308 | **0.002** |
| Ordinary | 0.029 | [-0.099, 0.156] | 0.443 | 0.669 |
| Poor | 0.144 | [-0.015, 0.293] | 2.189 | 0.056 |
| Physical health status |  |  |  |  |
| Good | -0.595 | [-0.781, -0.409] | -6.278 | **<0.001** |
| Ordinary | -0.110 | [-0.256, 0.036] | -1.483 | 0.172 |
| Bad | 0.706 | [0.454, 0.958] | 5.465 | **<0.001** |
| Native place | -0.100 | [-0.281, 0.081] | -1.081 | 0.308 |
| Only child | -0.104 | [-0.227, 0.016] | -1.704 | 0.123 |
| Smoking | 0.496 | [0.276, 0.716] | 4.416 | **0.002** |
| Drinking | 0.594 | [0.420, 0.768] | 6.706 | **<0.001** |
| Mental health status |  |  |  |  |
| Good | -0.853 | [-1.079, -0.627] | -7.397 | **<0.001** |
| Ordinary | 0.018 | [-0.070, 0.106] | 0.397 | 0.701 |
| Bad | 0.835 | [0.598, 1.072] | 6.916 | **<0.001** |
| Roommate sleeps late | 0.221 | [-0.078, 0.520] | 2.175 | 0.058 |
| Noisy dormitory | 0.627 | [0.420, 0.835] | 5.932 | **<0.001** |
| Dormitory light is bright | 0.036 | [-0.149, 0.221] | 0.382 | 0.711 |
| Poor dormitory hygiene | -0.046 | [-0.309, 0.216] | -0.345 | 0.738 |
| Romantic relationship status ever | 0.143 | [-0.235, 0.272] | 0.143 | 0.890 |
| Romantic relationship status being in love | 0.097 | [-0.033, 0.223] | 1.461 | 0.178 |
| Received psychological counseling | 0.746 | [0.568, 0.925] | 8.216 | **<0.001** |

Table S4 Model tuning configuration and results for XGBoost

| Parameter | Default | Constraint | Tunable | Best Value |
| --- | --- | --- | --- | --- |
| learning rate | 0.01 | - | TRUE | 0.01 |
| max_depth | - | 1, 20 | TRUE | 6 |
| min_child_weight | - | 1, 10 | TRUE | 3 |
| subsample | - | 0.5, 1 | TRUE | 0.8 |
| colsample_bytree | - | 0.5, 1 | TRUE | 0.8 |
| gamma | - | 0, 0.1 | TRUE | 0 |
| nrounds | - | 100, 300 | TRUE | 200 |
| Tuning AUC |  |  |  | **0.7185** |
| Tuning Accuracy |  |  |  | **0.7461** |

Table S5 Performance metrics and explanations for XGBoost

| Metrics | Training set | Test set | Full set | Explanation |
| --- | --- | --- | --- | --- |
| Accuracy | 0.7461 | 0.7321 | 0.7984 | Proportion of samples correctly predicted by the model |
| AUC | 0.7185 | 0.7063 | 0.8180 | Measure the model’s ability to distinguish classes |
| Precision | 0.5821 | 0.5479 | 0.6134 | Proportion of positive predictions that were actually correct |
| Recall | 0.1522 | 0.1652 | 0.2465 | Proportion of actual positive cases correctly identified |
| F1 | 0.2413 | 0.2539 | 0.3517 | Harmonic mean of precision and recall, balances both |


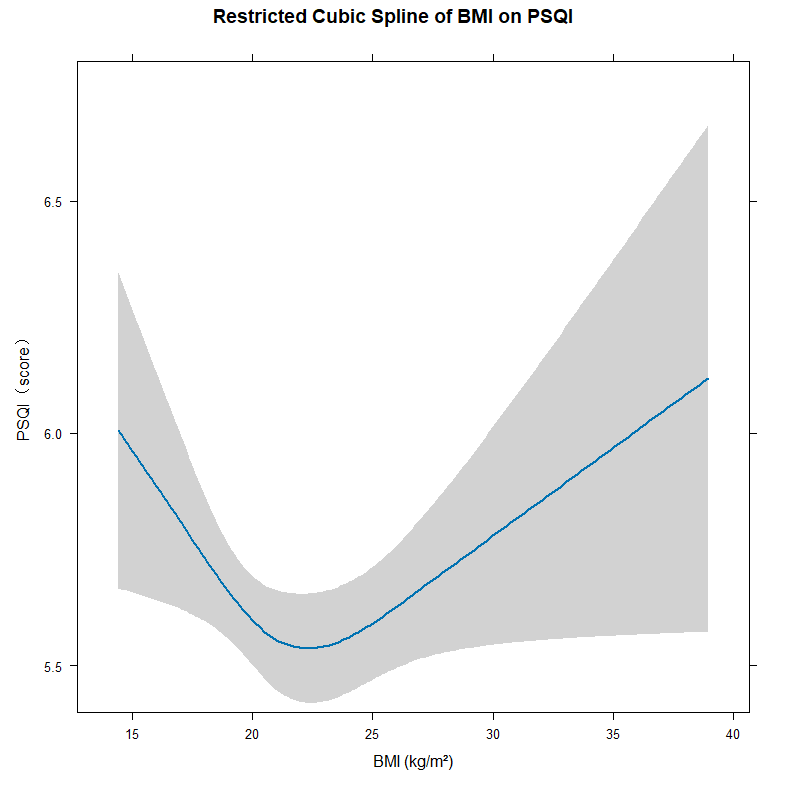


Figure S4 Restricted cubic splines for the nonlinear relationship between BMI and sleep quality


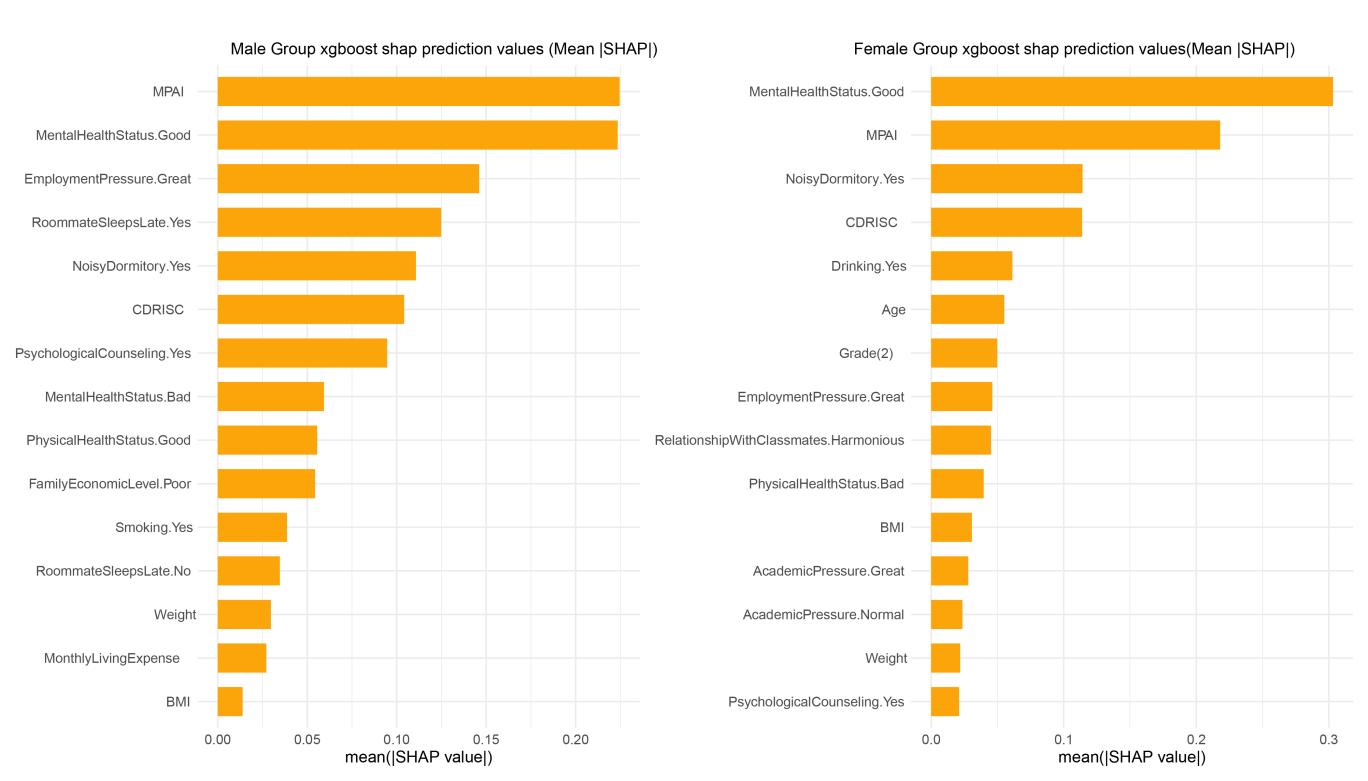


Figure S5 Comparison of factor importance orders in XGBoost across different gender groups


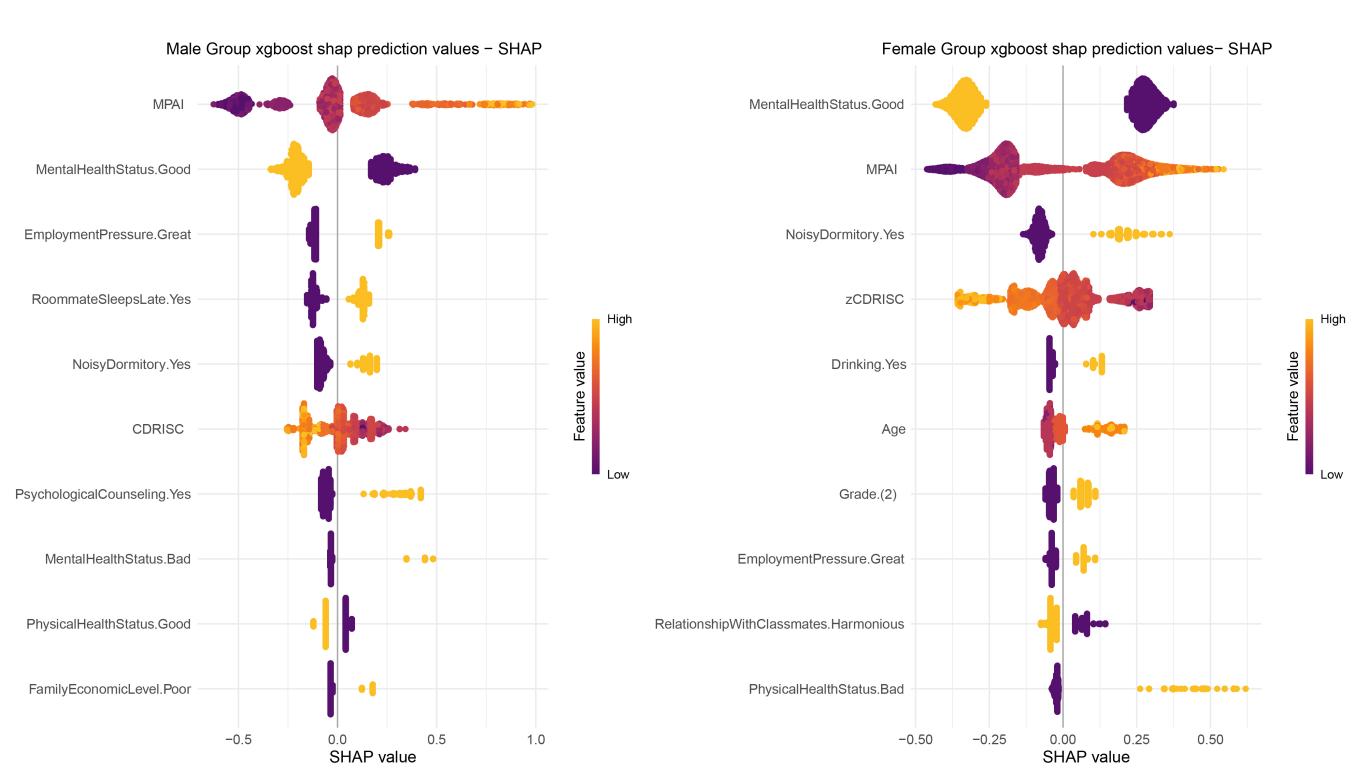


Figure S6 Comparison of SHAP swarm plots for XGBoost across different gender groups
